# Burden and Gender inequalities around Informal Care

**DOI:** 10.17533/udea.iee.v38n1e10

**Published:** 2020-02-26

**Authors:** Giuliana F. Cascella Carbó, Rosa García-Orellán

**Affiliations:** 1 Nurse, Servicio Navarro de Salud, , Pamplona Spain. MSc. Email: giuliana.cascella@gmail.com Servicio Navarro de Salud-Osasunbidea Servicio Navarro de Salud Pamplona Spain giuliana.cascella@gmail.com; 2 Nurse. Anthropologist. PhD. Professor, Universidad Pública de Navarra, Pamplona, Spain. Email: rosa.garcia@unavarra.es Universidad Pública de Navarra Universidad Pública de Navarra Pamplona Spain rosa.garcia@unavarra.es

**Keywords:** patient care, caregivers, gender and health, gender inequalities, gender perspective., assistência ao paciente, cuidadores, gênero e saúde, iniquidade de gênero, perspectiva de gênero

## Abstract

This work comes from the interest and need to understand the problems arising from the activity of caring for dependent people, in the world and particularly in the European region. Altogether, it seeks to understand the consequences of informal care on the caregiver adding to the debate a gender perspective. Through a multidisciplinary bibliographic review, the current care crisis becomes clear. The demographic and socio-cultural changes in recent years are causing dependency to increase dramatically, while putting at risk the availability of informal caregivers. Several studies have shown that women are the ones on whom the burden of care mainly falls. Therefore, under the gender perspective, it becomes clear that the consequences of caregiver burden increase gender inequalities worldwide. The study analyzes the current situation and underlines the need to promote alternatives and opportunities so that care is shared and does not fall only on the female gender. Solutions need to be included in public and community health interventions and policies, and to this respect, nurses play an important role in changing the care paradigm.

## Resumo

Este trabalho decorre do interesse e da necessidade de entender o problema dos cuidados informais globalmente e, em particular, na Europa. Além disso, busca entender as consequências do cuidado informal sobre o cuidador na perspectiva de gênero. Através de uma extensa revisão bibliográfica multidisciplinar, a atual crise de atendimento é revelada. As mudanças demográficas e socioculturais dos últimos anos aumentam a dependência cada vez mais. Isso leva a uma maior necessidade de cuidados. Vimos que a maioria dos cuidadores são mulheres e também que as consequências do cuidado aumentam as desigualdades de gênero em nossa sociedade. Este trabalho destaca a necessidade de promover alternativas e novas oportunidades de compartilhamento de cuidados e não apenas de uma pessoa. As respostas a essas necessidades devem ser incluídas nas políticas e intervenções em saúde e, nesse contexto, os enfermeiros desempenham um papel crucial na promoção dessas mudanças.

## Introduction

One of the biggest challenges for modern societies is the aging population, which together with the rise in life expectancy and the increase of chronic diseases, leads to a considerable growth of dependency worldwide.(1) Human beings are born without the capacity to survive in the absence of the care of another person and this characterizes the intrinsic fragility of all.(2) Care is necessary especially during vulnerable periods of life, like childhood, illness, disability and senility.(3,4) The act of care has mitigated this fragility through human history and it has traditionally been covered within the family setting by women.(5) However, socio-political changes such as the transformations in the family structure(6,7) and the increased participation of women in the public sphere including the labor market,(8) have disturbed the capacity of families to provide the care needed by its elder or disable members.

We are therefore witnessing a growing “care crisis”,(9,10) where it becomes urgent to face the challenges that arise when talking about the needs of the disable and elder. It is in this context of “care crisis” where the debate on informal care gains importance(11) and it becomes urgent to include a gender perspective to analyze the situation.(12) Aging population, illness, disability, and dependency are increasing globally and 80-90% of care needed is provided domestically by informal careers.(11,12) Thus, health systems depend upon informal care(5) as they cannot fully cover the care services needed. However, the capacity of informal caregivers to provide for such care is being undermined. 

Along with social transformations, the consequences of informal care on the caregiver itself are also to be considered. Several studies indicate that informal care is delivered in most cases by a single person.(13-15) This circumstance makes the burden of caring so high that informal careers can suffer extreme physical and psychological consequences resulting from the lack of support in the caring duties, affecting negatively on their health and quality of life.(16,17) All these factors contribute to destabilizing the family solidarity upon which the current care system is based(18,19) and undermine the availability of informal care for present and future generations.

Nowadays it is widely recognized that the figure of the informal caregiver is crucial for the wellbeing of people with dependency. Along with this awareness, the literature on informal care has grown significantly in the last four decades (20). However, the mainstream approach has considered the caregiver instrumentally, as a tool to obtain the main goal, improving the health of the dependent person.(21) Only more recently, new approaches have started to focus on the caregiver not only as a provider but also as a client of care(21) and to consider the family as a whole, instead of focusing only on the caregiver.(22) Also, attention has been given to the economic and time costs of informal care to raise awareness on how these factors impact health systems.(23-25) Finally, gender inequality in informal care is emerging as a novel subject of investigation (8). This article underlines the importance of the gender perspective in studies regarding informal care and supports the notion that considering gender inequalities is necessary to fully understand the care crisis and design policies and interventions aimed at promoting the sustainability of informal care.

In the traditional care model, women take the biggest share of unpaid care work, being women around 80% of informal caregivers worldwide (14), with similar numbers in Europe.(9,12,15,26) The unequal distribution of care and domestic responsibilities between women and men reinforce the persistent gender inequalities in the family and working spheres (9). It is important to consider the issue of informal care from a gender perspective, to identify sustainable and accurate responses.(8,27) 

The care crisis is a problem of Public Health and a relevant challenge for the XXI century nursing.(8,28) As WHO states in its World Report on Ageing and Health,(11) the challenge of the demographic transition to older populations is not taken seriously and “care and support for caregivers…is not a priority focus of government action on aging”.(11^,^^18^) Therefore, professional and skilled caregivers, the nurses, have to quickly adapt to the social transformations to be able to provide appropriate answers to the problem. Care and support for informal caregivers and their families have to be the prior objective of both, governmental action and the nursing profession. For community nursing this challenge could be an opportunity to get stronger, improve its competencies and focus on the needs of patients, families and the community, to ultimately support other agents of care as informal caregivers.(29)

This work aims to comprehensively review the literature on informal care, caregiver burden and its relationship with gender inequalities in the fields of nursing, health, cultural and gender studies. It aims to highlight the importance of including a gender perspective in the debate about informal care to fully understand the situation and be able to give appropriate responses. 

## Methods

Bibliographic review on informal care, starting from the global and focusing on the regional reality of Europe and Spain. Databases: Web of Science, PubMed, Cuiden, and Dialnet. The searches used the following searching criteria: 1) Keywords: “informal care”, “caregiving burden”, “dependency”, “family caregiver”, and in Spanish “cuidado informal”, “cuidadores informales”, “dependencia”, “mujeres”, “genero”, “sobrecarga del cuidador” “síndrome del cuidador”. 2) Publication date range: 2000-2019, however, some fonts older than 2000 were included for their relevance. 3) Languages: Spanish, English. 

To follow a multidisciplinary perspective, articles from various disciplines were analyzed and included: health science, especially gerontology and nursing, sociology, anthropology, gender studies, cultural geography, cultural studies. 

Articles excluded: studies about informal care in specific pathologies, studies analyzing the situation of informal care outside the geographical areas of interest. Qualitative studies were revised to understand the social and cultural aspects of informal care and to include the gender perspective. Quantitative research studies were also included to understand the characteristics of informal caregivers and the impact on their health.

Epidemiological and statistical data were consulted to understand the local situation of dependency, aging, and informal caregivers. 

## Results

### Gender and Care

“To care is currently a very important verb, and contemporary societies […] assign it as a natural condition to the female gender; in this way, it is the women who take care of other’s vital needs: children, family, ill people, grandparents, grandchildren.”(30, p 119) But is care intrinsically feminine? Why informal caregivers are mainly women? Is it cultural? Caring seems to be a predominantly women’s activity and its study seems to require an analysis rooted in the gender order.(4) But is caring something feminine by nature? The social organization of care activities and the place they occupy in today's society are the product of a long historical process that began to take shape during the transition to liberal capitalism.(31^,^^32^)

In western societies, impregnated with Christian values, women have traditionally dedicated themselves to the family and the reproductive sphere, while men have had a greater participation in the productive and public sphere. The responsibility of care in general, dependency, childhood, old age, home care, etc., has been restricted to the private or domestic space, the reproductive sphere assigned to women.(30,32) In recent years, a gender perspective has been introduced in the study of care. Including this perspective, care ceases to be attributed to the universe of the feminine, in an "essentialist or naturalistic way”,(44, p45) becoming the "social and historical conditions of such naturalization" (*Ibid*). In this context, Carol Gilligan's work is fundamentally important, since in her *Ethics of Care,*(34) she insists that the fact that care is “feminine” is part of a social construction and learning throughout our lives, since childhood, of a specific ethic. In addition to social and historical construction, there is also an emotional component of care related to the fact that the act of caring is often satisfactory. It is not yet clear if this emotional element is what makes women to care the most, generating debate in the fields of ethics and moral philosophy.(4)

Besides the philosophical debate, the literature in informal care tell us that today it is women who take the most responsibility in the care for dependency in the world.(26,35,36) Despite the socio-cultural changes of the last 30 years that led to a greater participation of women in the labor market, and a slight increase in the participation of men in the domestic work, including childcare,(8) there are still major gender inequalities both in the field of reproductive work, in terms of care, home, children and dependents, and in the field of productive work.(37) The fact that care has been, and continues to be considered a matter of the feminine sphere, reinforces gender stereotypes about the roles of men and women in the society.(7)

### Informal Care and the Care Crisis

Globally, the population is getting older,(11) and this phenomenon is no longer affecting only high-income countries, but it is also a reality in low- and middle-income ones.(38) As a result, the global population aged 60 years or older is increasing significantly. Predictions suggest that by 2050 the percentage of elders, aged over 60, will reach 30% of the population in Europe, North America, China, Chile, and other largely populated countries.(11) This global phenomenon is caused by the reduction of mortality around the world together with an increase in life expectancy and falling fertility rates.(11) Moreover, this increase in life expectancy often associates with more dependency throughout those added years,(39,40) as chronic diseases and multimorbidity grow.(11) Indeed, conditions such as heart disease, dementia, chronic respiratory disorder, stroke, diabetes, and some musculoskeletal conditions are the major causes of disability for people aged over 60 and are all increasing globally.(38) It is estimated that worldwide there are 349 million people care-dependent, defined as the condition when individuals are no longer able to undertake basic daily living tasks alone.(38) As the number of care-dependent people grows, the need for informal care providers also increases.

Informal care is defined as the type of unpaid care of people with different grades of dependency, normally, but not always, provided by family members.(1,7,11,29) This kind of care accounts for 80-90% of dependency care (12,29) and it is usually done at home. The availability of informal caregivers is at risk worldwide and the care crisis takes its characteristics in each region and even in each country. In the European region, the situation of informal care has both advantages and disadvantages compared to other regions of the world.(13) On one side we find that the majority of the countries in the region are considered as “high or middle-high income”, following the WHO classification.(11,38) This translates to better coverage of health systems, a bigger range of public services allocated to help dependency and in general a greater development of the Welfare State.(5) On the downside, we have the demographic trends and socio-cultural changes occurring in the family structures.(15,41) The aging of the population and the growing demand for health and social services for dependent people put at risk the sustainability of the European Welfare State since the state is usually the main provider of such services.(15) Health spending grows faster than the Gross Domestic Product (GDP),(13) another reason to recognize, support and strengthen informal care as a fundamental part in the present and future of the care needs of the European population (*Ibid*.).

Europe has today the eldest population of the world, and it is estimated that by 2050 more than 30% of its population will be over 60 years old.(11) This alone is a big challenge for European societies. Besides, social and demographic changes have modified the family structures. While in rural areas the traditional family survives, in which many generations live together and family members take care of each other, in the urban areas, where most of the European population lives, the situation is very different.(13) In the cities, family units are getting smaller, family members disaggregate and spaces are limited for the cohabitation of several generations. Besides, we are witnessing a ‘verticalization’ of the family, i.e. the increasing life expectancy allows more generations to coexist longer increasing vertical family relationships (children-parents-grandparents).(18) All the reasons mentioned suggest that the elder of tomorrow will need more care and that families will be unable to provide it.(13) Though not everything is negative, the verticalization of the family also brings new opportunities for exchange and intergenerational solidarity.(18)

Social changes make it more difficult for old and dependent people to stay in their homes due to the lack of family support. However, it has been shown that home and community are the ideal places for the life and care of the elder and significantly improve their health and quality of life.(1,42) Not only is home and community-based care preferable over institutionalized care because of its benefits for health and quality of life,(11,42) but it is also preferred by the older people and their families.(12,14) Today the proportion of informal and formal care varies from country to country, influenced not only by social policies and the state's degree of responsibility for long-term care of dependency, but also by family structures, levels of intergenerational assistance, and cultural norms about care.(13) The proportion of informal caregivers in the different European states is between 20% and 44% of the entire population.(15) For intensive caregivers, defined as those persons that dedicate more than 11 hours per week to informal care, the percentage varies between 4% and 11%.(13,15)

In general, in the countries of northern Europe, formal home care has developed considerably in recent years, this for several reasons. For instance, in such countries, socio-cultural changes have been faster, higher income levels and greater economic capacity are present both in health systems and in the population, altogether allowing for a greater share of formal and informal paid care.(15) Therefore, formal care covers a large part of home and community assistance, although informal care of family and friends continues to cover most of the psychological and emotional needs of dependent people.(13) Conversely, in countries in southern and eastern Europe, informal care covers the largest proportion of assistance, both physical and psycho-affective.(13) 

Contrary to what one might think, the proportion of informal caregivers in northern Europe is higher than in countries of southern and eastern Europe.(15) In these countries, compared to northern Europe, the proportion of intensive caregivers is higher.(15,41) This means that more people dedicate more than 11 hours per week to informal care duties and suggests that in those countries where the State does not support assistance and leaves the care responsibility to the families, there are fewer people willing to do it and those that end up doing it, do it with a greater intensity.(15) This is a key point when talking about "caregiver burden" and gender inequalities.

Women between 45 and 60 years old are the main informal care providers in all European countries.(12) If we consider intensive caregivers, there are also women the majority, with a higher percentage in southern countries.(30) Moreover, in the European region, the social changes previously mentioned, are further accentuated. For instance, women’s participation in the labor market has risen considerably.(15,30) In countries where strong policies to encourage the participation of women in the labor market are in act, the implications for the availability and provision of informal care are enormous.(13)

### The Impact of Caregiving with a Gender Perspective

The literature on caregiver burden is extensive. It has been decades since the problem has been identified. It is well known that caregivers suffer a physical, psychological and emotional burden. They are not only responsible for medication, hygiene, and food administration, but provide also emotional support and, on many occasions, are responsible for taking important decisions for the person cared for.(43) Caring for dependents means an important dedication of energy and time. According to some studies, 95% of caregivers of people 65 and older refer to dedicate 6 to 7 days a week to care activities, and 38.9% refer to dedicate at least 16 hours a day to care.(29) Besides, considering that the degree of disability usually increases over time, the time needed to care growths accordingly, leading to a gradual loss of independence of the caregiver, which ends up paralyzing or postponing their life project.(43) The negative impact on the quality of life of the caregiver is enormous and it affects various spheres, such as health, relationships, self-care, and economy.

Knowing that the majority of caregivers are women,(44) it can be affirmed that they are those who suffer most of the consequences and the burden of informal care.(8,45) It is interestingly to note that not only women caregivers are more, but they also suffer the burden differently than men. In fact, by comparing women and men caregivers, it has been reported that women are those that suffer the worst consequences of the care burden(46) in all the spheres: health, economic and personal relations, including self-care and family. This, also in part due to existing gaps between the two genders.(28) 

In terms of health, the impact of care overload on one's physical and mental health is enormous: in a study conducted in 2008 in Spain, 32.7% of caregivers reported fatigue, 27.5% reported that their health had deteriorated and 18.1% felt depressed.(18) Among the most common physical complaints are fatigue, musculoskeletal pain, stress, insomnia and headaches (*Ibid.*).

It is known that women’s health differs from that of men.(45) While life expectancy is higher in women, health surveys have identified more chronic problems and worst perceived health for them.(45,47) Regarding caregivers, it has been observed that women are more affected by the burden of care than men.(26) In several surveys, women declared more fatigue and physical conditions, as well as depression; also, many of them reported having to take medications to handle the overload situation.(18) This is in part because tasks performed by women are often different from those performed by men caregivers. For instance, male children caregivers usually dedicate more to tasks such as making arrangements, while caring daughters are more concerned about hygiene and daily living activities.(48) 

Dependent care not only affects physical health but also the psycho-affective sphere. Data vary according to reports, for example, around 50% of caregivers refer that caring has caused them mood swings, even altering their personality, 77% of whom consider these changes as "considerable alterations".(43) Among the symptoms referred to as psycho-affective alterations, there are frustration and helplessness (73%), anxiety (61.5%), depression or sadness (57.5%), loneliness (35%), guilt (30.5%) and irritability or anger (60%).(43) Here also women seem to be more affected than men; commitment and emotional involvement are usually greater in women caregivers,(18) which leads to greater health problems in the psycho-affective sphere.

Another area in which the caregiver is severely affected is the sphere of family life, self-care, and leisure time. Regarding family, the burden of informal care negatively affects social relationships and creates family tensions. It breaks family solidarity, which could be ultimately lost. It has also been reported that many caregivers devote more time to the care of the dependent person than to the care of their children, while in terms of self-care and leisure time, according to a survey on informal care in Spain, 61% of caregivers report having had to reduce their leisure time, 27% do not have time to take care of themselves and 17% do not have time to take care of other people.(18) The negative consequences of caring are worsened by gender differences in the sphere of personal life. In general, women practice less physical exercise, sleep fewer hours and enjoy less leisure time than men, commonly because of the care responsibilities they usually have in the family environment.(49) If we add to these differences the care of a dependent relative, the gap accentuates even more. Besides, it has been observed that women and male caregivers receive different support from the family, with, once again, women receiving less family support, while male caregivers receive more collaboration.(35)

In addition to the consequences on health, time and family relationships, in the area of professional and paid work-life, known as the *productive sphere,*(37) there are also important effects. Among the economic consequences are: not being able to work outside the home, having had to leave work or to reduce working hours, having problems at work due to difficulties in meeting schedules or not being able to go to work in emergencies.(18) Moreover, it has been estimated that people who are dedicated to care have fewer opportunities to find work, a higher risk of leaving their studies, more chances of having to reduce their working hours or having to ask for unpaid leave to care, are ultimately are more likely to stop working and to retire earlier.(24)

All this is often not taken into account in studies about the caregiver's burden, although the economic aspect is of fundamental importance since it accentuates health, socioeconomic and gender inequalities.(50) In the case of women, being an informal caregiver sums up to existing gender inequalities in wages and opportunities.(8) It is clear then that women caregivers are more affected than men in their professional careers, in their income and, as a consequence, also in their tax and retirement rights.(7,18,51) Although the employment rate among women has increased progressively in recent years, it is still more frequent for women to reduce their workday or leave their work to devote themselves to care for dependent family members,(18) as with childhood care. For example, in 2011 in Spain, 93% of the total leave permits for informal care of children and dependent relatives were requested by women.(52) The increased participation of women in the labor market has not been accompanied by an equitable distribution of reproductive work and domestic labor. More frequently women are therefore affected by the “double day” consequences, where they cover the responsibilities of their paid work keeping the responsibilities of the domestic chores.(33) If we add to this, the care of a family member, the burden becomes an unsustainable “triple burden”.

Having said that, it can be argued that the unequal distribution and burden of informal care between genders is based on, and at the same time increases gender inequalities in health and ultimately in society. There are several important reasons to address gender inequalities in health. For instance, gender inequalities together with socioeconomic status are the major causes of inequalities in health, including those related to the availability and use of health resources and services.(53) More interestingly, evidence suggests that by incorporating a gender perspective in health policies, plans and programs, health inequalities can be reduced and the effectiveness and efficiency of health services improved.(54)

Some studies have identified gender biases in health care that usually impair women in areas such as diagnostic, therapeutic effort and health research.(55-57) It has also been observed that in Primary Care, women are asked less about their lifestyle than men, which limits the equal benefit of the scope of health prevention and promotion activities that are carried out daily in health centers.(47) Studies have shown that the perceptions of health professionals towards care have a big impact on how they take care of the caregiver. Sometimes those precocious and attitudes result in interventions that are potentially negative for gender equity; an example is the conservative attitude that gives the family the main responsibility for caring and some sexist stereotypes that give women more abilities to do it.(58) Interventions to address dependency related problems, such as the caregiving burden, should take into account gender inequalities.(48) Several studies emphasize that it is necessary to develop interventions and strategies that do not reinforce gender roles in informal care, but rather encourage better and greater distribution of care tasks among more people, men and women.(35) Among those interventions, it becomes necessary to train health professionals on gender equity, as a tool to reduce gender gaps in informal care to improve the quality of life of informal caregivers and people cared to.(58) Unfortunately, addressing gender inequalities in health in plans, programs, and interventions, has not been so common(5,29,37) and more research is needed on the subject. 

Thus, to address the care crisis, collectivization of care is necessary through collaboration between state institutions, the market, civil society, and families, to build complementary and beneficial relationships for all parties.(59) Finally, it is worth mentioning that the role of men in care is also changing. In Latin America and in other regions where migratory trends have changed family structures, women have had to migrate in search of paid work, so men have had to take care of children and dependent family members, changing traditional roles of women in care.(60) Several studies show how this has affected men, who, when women in the family are not present or available, assume the role of informal caregivers, breaking gender stereotypes and also changing their perception of themselves.(26,61,62) From this new trend, new masculinities emerge(32) which reminds us that family roles and gender identities are not immutable or universal but change and adapt to new needs.(60) 

## Discussion

After reviewing the bibliography on the issue of informal care, we have come to know the status of the issue of informal care both globally and in the European region. Likewise, we have seen that the care crisis(33) is a growing problem worldwide and that this can cause the pillars of the welfare system to collapse if this crisis is not properly addressed.(63) All of the foregoing underlines and supports the hypothesis that, in today's society, vulnerability and dependency can no longer be considered as exceptional situations in people's lives, but rather are intimate characteristic of the human condition inherent in the existence of anyone.(4) That is why informal care is a central issue for the health of the population and as the aforementioned socio-demographic changes are taking place, it is becoming increasingly urgent to address this problem in health and social welfare public policies.(5)

We face two different but intimately related issues: informal care and gender inequalities in health. The evidence shows that one of the keys to dealing with the aging of societies and dependency care is the creation of primary care programs that include community services and support for families and caregivers.(38) But this cannot be possible if a gender perspective is not taken into account, since, as we have detected in the revised bibliography, the burden of care falls mostly on women and this triggers gender inequalities in health to grow. Gender inequalities in health add up to other gender inequalities present in our society.

If we consider that the way care is delivered is a social and historical construction,(34) and the current care model is in crisis, deconstruct the existing patterns of care is necessary to reconstruct a new model in which care is no longer delivered by a single person, a women, but it is shared by all family members with the support of institutions and the civil society. A better distribution of care responsibilities between women and men becomes every day more urgent and necessary.(9) Nurses, especially community nurses, are the health professionals that are closer to informal caregivers and include them in community intervention programs, individual assessment as well as prevention and health education. The scientific literature in the nursing field on informal care has focused on health problems affecting the caregivers and interventions centered on promoting self-care to avoid caregiver overload.(29) As the literature revised suggest, this is no longer sufficient to address the current care crisis. The practice of nursing care could contribute enormously to the sustainability of the care system.(64) In other to achieve this, a gender perspective should be included in both, theoretical analysis and professional practice. Including a gender perspective in nursing professional practice could help eradicate gender inequalities in health,(28) and the ambit of informal care is a great opportunity to make this.

The conclusion of this review is that the care crisis is a global problem that is reflected in the local realities. The system of informal care based on the almost exclusive participation of women, with all the negative consequences that this entails, is unsustainable. Including a gender perspective in the development of intervention plans to help caregivers improve their quality of life is desirable and necessary. Breaking with the socially designated roles of women in care can be the change that allows a different balance in the work of caring, which could improve the quality of life of dependent people, caregivers and their families throughout society.

Primary Care, and in particular community nursing, from its privileged position of closeness to people and the community, can be the engine of the change of the traditional model of care, for this, it is necessary to include the gender perspective in nurse practice and especially in analysis and interventions aimed at caregiver care. To address the crisis of care, collectivization of care is necessary through collaboration between state institutions, the market, civil society, and families.
